# Supplementary material for: Transcriptional Profiling of Immune and Inflammatory Responses in the Context of SARS-CoV-2 Fungal Superinfection in a Human Airway Epithelial Model
Source: Microorganisms. 2020 Dec 11;8(12):1974. doi: 10.3390/microorganisms8121974 (PMC7764715; doi:10.3390/microorganisms8121974)
Supplement: Supplementary file 1 [file microorganisms-08-01974-s001.zip › microorganisms-1006524-supplementary.docx]

**Supplementary Materials**

**Figure S1.** Complete view of functional enrichment results for SARS-CoV2 or SARS-CoV-2+Aspergillus infected conditions vs. Mock. Considering CoV vs. Mock and CoV+Asp vs. Mock for both bronchial and nasal epithelium type. significantly up- or down- regulated gene lists (x-axis) were tested for significant enrichment using the parent-child strategy (see methods). If below the threshold (0.05). The adjusted *p*-values corresponding to different terms (y-axis) are represented by point sizes (see legend). Terms were clustered based on gene occurrences (binary distance & Ward algorithm) in 15 metagroups. The bar plot on the right represents the sizes of enriched terms (called child) in comparison to the size of their parents (see methods for definitions).

**Figure S2.** Complete view of functional enrichment results of SARS-CoV-2+Aspergillus superinfection vs. SARS-CoV2 infection. Considering CoV+Asp vs. CoV for both bronchial and nasal epithelium type. significantly up- or down- regulated gene lists (x-axis) were tested for significant enrichment using the parent-child strategy (see methods). If below the threshold (0.05). the adjusted *p*-values corresponding to different terms (y-axis) are represented by point sizes (see legend). Terms were clustered based on gene occurrences (binary distance & Ward algorithm) in 10 metagroups. The bar plot on the right represents the sizes of enriched terms (called child) in comparison to the size of their parents (see methods for definitions).

**Supplementary Data File 1.** Complete list of deregulated genes in SARS-CoV2 or SARS-CoV-2+Aspergillus infected conditions vs. MocK

**Supplementary Data File 2.** Complete list of deregulated genes in SARS-CoV-2+Aspergillus superinfection vs. SARS-CoV2 infection.

**Table S1.** List of URLs parsed to build the databases used for enrichment analysis. Three types of information were used (i) details about terms meaning term ID. complete name and contingent category (e.g Biological process or Molecular function for Gene Ontology); (ii) gene-term associations; (iii) topology and especially child to parent’s relations to apply the parent-child method. .

| Database | Information type | URLs |
| --- | --- | --- |
| Reactome | Details (name.Id) | https://reactome.org/download/current/ReactomePathways.txt |
| Reactome | gene-term associations | https://www.uniprot.org/uniprot/?sort=score&desc=&compress=no&query=organism:%22Homo%20sapiens%20[9606]%22&fil=&format=tab&force=yes&columns=id.entry%20name.length.genes(PREFERRED).reviewed.database(Reactome) |
| Reactome | Topology | https://reactome.org/download/current/ReactomePathwaysRelation.txt |
| Uniprot Keywords | Topology | http://ftp.ebi.ac.uk/pub/databases/interpro/ParentChildTreeFile.txt |
| Uniprot Keywords | Details (name.Id) | http://ftp.ebi.ac.uk/pub/databases/interpro/entry.list |
| Uniprot Keywords | gene-term associations | https://www.uniprot.org/uniprot/?sort=score&desc=&compress=yes&query=organism:%22Homo%20sapiens%20[9606]%22&fil=&format=tab&force=yes&columns=id.entry%20name.length.database(InterPro).genes(PREFERRED).reviewed |
| Gene Ontology | Topology and Details (name.Id) | https://www.uniprot.org/keywords/?sort=&desc=&compress=yes&query=&fil=&format=obo&force=yes' > keywords/uniprot_keywords.obo.gz |
| Gene Ontology | gene-term associations | https://www.uniprot.org/uniprot/?query=organism%3A%22Homo%20sapiens%20%5B9606%5D%22&sort=score&columns=id%2Centry%20name%2Ckeywords%2Cgenes(PREFERRED)%2Cdatabase(GeneID)%2Creviewed&format=tab |

**Table S2.** Statistics of RNA-Seq fragment pseudo-alignment to the human transcriptome.

| Epithelium type | Sample | Percentage of fragments aligned | Millions of fragments aligned |
| --- | --- | --- | --- |
| Bronchial | CoV+Asp | 22.30% | 9.6 |
|  | CoV+Asp | 25.60% | 5.7 |
|  | CoV+Asp | 25.20% | 7.1 |
|  | CoV | 61.50% | 13.8 |
|  | CoV | 66.30% | 15.9 |
|  | CoV | 46.00% | 11.5 |
|  | Mock | 80.30% | 19.1 |
|  | Mock | 78.60% | 20.9 |
|  | Mock | 79.20% | 18.5 |
| Nasal | CoV+Asp | 45.40% | 10.8 |
|  | CoV+Asp | 47.00% | 15.1 |
|  | CoV+Asp | 40.90% | 9.5 |
|  | CoV | 53.00% | 15.2 |
|  | CoV | 53.00% | 14.4 |
|  | CoV | 57.10% | 15.7 |
|  | Mock | 82.20% | 21.7 |
|  | Mock | 80.70% | 22.1 |
|  | Mock | 80.40% | 19.2 |
